# Change of metformin concentrations in the liver as a pharmacological target site of metformin after long-term combined treatment with ginseng berry extract

**DOI:** 10.3389/fphar.2023.1148155

**Published:** 2023-03-10

**Authors:** Choong Whan Lee, Byoung Hoon You, Sreymom Yim, Seung Yon Han, Hee-Sung Chae, Mingoo Bae, Seo-Yeon Kim, Jeong-Eun Yu, Jieun Jung, Piseth Nhoek, Hojun Kim, Han Seok Choi, Young-Won Chin, Hyun Woo Kim, Young Hee Choi

**Affiliations:** ^1^ College of Pharmacy and Integrated Research Institute for Drug Development, Dongguk University_Seoul, Goyang-si, Gyeonggi-do, Republic of Korea; ^2^ National Center for Natural Products Research, School of Pharmacy, The University of Mississippi, University, MS, United States; ^3^ Department of Rehabilitation Medicine of Korean Medicine, Dongguk-University Ilsan Oriental Hospital, Goyang-si, Gyeonggi-do, Republic of Korea; ^4^ Division of Endocrinology and Metabolism, Department of Internal Medicine, Dongguk University Ilsan Hospital, Goyang-si, Gyeonggi-do, Republic of Korea; ^5^ College of Pharmacy and Research Institute of Pharmaceutical Sciences, Seoul National University, Seoul, Republic of Korea

**Keywords:** metformin, ginseng berry extract, pharmacokinetics, tissue distribution, organic cation trans porters, multidrug and toxin extrusions

## Abstract

Metformin as an oral glucose-lowering drug is used to treat type 2 diabetic mellitus. Considering the relatively high incidence of cardiovascular complications and other metabolic diseases in diabetic mellitus patients, a combination of metformin plus herbal supplements is a preferrable way to improve the therapeutic outcomes of metformin. Ginseng berry, the fruit of *Panax ginseng* Meyer, has investigated as a candidate in metformin combination mainly due to its anti-hyperglycemic, anti-hyperlipidemic, anti-obesity, anti-hepatic steatosis and anti-inflammatory effects. Moreover, the pharmacokinetic interaction of metformin *via* OCTs and MATEs leads to changes in the efficacy and/or toxicity of metformin. Thus, we assessed how ginseng berry extract (GB) affects metformin pharmacokinetics in mice, specially focusing on the effect of the treatment period (i.e., 1-day and 28-day) of GB on metformin pharmacokinetics. In 1-day and 28-day co-treatment of metformin and GB, GB did not affect renal excretion as a main elimination route of metformin and GB therefore did not change the systemic exposure of metformin. Interestingly, 28-day co-treatment of GB increased metformin concentration in the livers (i.e., 37.3, 59.3% and 60.9% increases *versus* 1-day metformin, 1-day metformin plus GB and 28-day metformin groups, respectively). This was probably due to the increased metformin uptake *via* OCT1 and decreased metformin biliary excretion *via* MATE1 in the livers. These results suggest that co-treatment of GB for 28 days (i.e., long-term combined treatment of GB) enhanced metformin concentration in the liver as a pharmacological target tissue of metformin. However, GB showed a negligible impact on the systemic exposure of metformin in relation to its toxicity (i.e., renal and plasma concentrations of metformin).

## 1 Introduction

The combination therapy of anti-diabetic drug with herbal products (i.e., complementary and alternative medicines) is practiced in over 30% of diabetic patients in the United States and more common in Asia (Wang et al., 2013; Gupta et al., 2017). Clinically, the use of a combination of antidiabetic drug with herbal medicines has already been proven to improve therapeutic outcomes. Reports and researches to evaluate the efficacy and/or safety for the drug combination with herbal products have been increasing (Wang et al., 2013; Wang et al., 2018; Yang et al., 2019). Moreover, the pharmacokinetic evaluation of herb-drug interaction (HDI), which is well-known among drug interactions, identifies how the herb changes efficacy and/or toxicity of a drug (Chen et al., 2012; Choi, 2020; Choi and Chin, 2020). The combined treatment of the herb can affect the circulating level and specific target tissue level of a drug, therefore altering the clinical outcome of the drug (Chen et al., 2012). To prevent unexpected risk or potential of HDIs, *in vitro* and *in vivo* models have been regularly adjusted and recommended to use in the evaluation of pharmacokinetic HDIs (Iwatsubo, 2020; Sudsakorn et al., 2020). Considering that many herbs are orally administered with a chronic regimen, the HDI evaluation is focused on their interactions in the intestine, liver, kidneys and pharmacological target organs (Chen et al., 2012; Choi, 2020; Choi and Chin, 2020).

Metformin, an oral glucose-lowering drug, is commonly used for the treatment of type 2 diabetic mellitus (Choudhary et al., 2012). Despite the relative safety and popular utilization of metformin for a long time, it has still been identified that mechanism of actions of metformin targeting multiple pharmacological sites in the body (Hermann et al., 1994; El-Mir et al., 2000; Madiraju et al., 2014). Individual variations of diabetic patients’ responses during metformin treatment have been sometimes observed (Hermann et al., 1994), and patients with diabetic mellitus shows the increased risks of cardiovascular complications and other metabolic diseases. Thus, combinations of metformin with herbal products are popularly conducted, and drug interactions of metformin with herbal products have been occasionally occurred (Wang et al., 2018; Yang et al., 2019). In terms of pharmacokinetics, metformin is well absorbed, and extensively distributed to the liver as its pharmacological target tissue and kidneys as its main elimination organ (Stage et al., 2015). In other words, metformin extensively distributed to liver, and reduces glucose production in the livers. Metformin is also considerably distributed to the kidneys and excreted into urine, which supports the idea that the renal excretion of metformin highly determines its systemic exposure and toxicity (e.g., lactic acidosis) (Gong et al., 2012). Thus, it is important to understand the transporter-mediated metformin disposition [i.e., organic cation transporters (OCTs) and multidrug and toxin extrusions (MATEs)] in livers and kidneys to elucidate the efficacy and toxicity of metformin, which has attracted attention in understanding metformin pharmacokinetics and pharmacodynamics (Sundelin et al., 2020; Yang et al., 2021).

Ginseng berry, i.e., the fruit of *Panax ginseng* Meyer, has been shown to ameliorate hyperglycemia, hyperlipidemia, obesity, hepatic steatosis and inflammation, mainly due to the pharmacological activities of the ginsenosides in ginseng berry (Dey et al., 2003; Zhang et al., 2016; Chae et al., 2019). Choi et al. (2018) proved that the combinated ginseng berry extract (GB) improved metformin’s glucose lowering effect without any adverse effect in clinical study, which accelerating the combination therapy of GB and metformin. Chae et al. (2019) reported that GB enhanced metformin efficacy against obesity and hepatic steatosis through AMPK activation. Therefore, as an extended follow-up study, the present work assesses how GB affects OCTs and/or MATEs-mediated changes on metformin pharmacokinetics. The treatment period effect of GB and metformin was specially evaluated in mice, because the combination of metformin and GB has the possibility to be applied in long-term treatments.

## 2 Material and methods

### 2.1 Chemicals and reagents

GB from 4-year-old Korean ginseng berries (*P. ginseng*) was provided from Amorepacific Corporation (Gyeonggi-do, Republic of Korea) (Chae et al., 2019). Metformin hydrochloride, carbamazepine [Internal standard (IS) for the analysis of liquid chromatography-tandem mass spectrometry (LC-MS/MS)], and cimetidine were purchased from Sigma-Aldrich Chemical Company (St. Louis, MO, United States. HEK293 cells overexpressing hOCT1 (SLC22A1), hOCT2 (SLC22A2), hMATE1 (SLC47A1) and hMATE2-K (SLC47A2) were purchased from Corning Life Sciences (Corning, NY, United States). Trizol and SYBR green supermix were purchased from the Molecular Research Center (Cincinnati, OH, United States) and TAKARA (Kusatsu, Shiga, Japan), respectively. All other chemicals and reagents were categorized as analytical grade.

### 2.2 Effect of GB on metformin uptake in HEK293-cells overexpressing hOCT1, hOCT2, hMATE1, or hMATE2-K

The effect of GB on metformin uptake was assessed using slightly modified methods (You et al., 2018). HEK293 cells overexpressing hOCT1, hOCT2, hMATE1 or hMATE2-K were seeded in 24-well plates coated with poly-D-lysine at a density of 4.0 × 10^5^ (cells/well and incubated for 24 h with Dulbecco’s modified eagle medium (DMEM) plus 10% fetal bovine serum (FBS). Cells were maintained at 37°C in humidified atmosphere 8% CO_2_. For cells overexpressing hMATE1 and hMATE2-K, DMEM supplemented with 10% FBS and 2 mM sodium butyrate was used instead of DMEM with 10% FBS. After washing the cells twice with prewarmed Hank’s balanced salt solution (Hank’s solution) with Ca^2+^ and Mg^2+^, the cells were preincubated with Hank’s solution for 10 min. To set the driving force of hMATE1 and hMATE2-K, the following step was conducted after preincubation: rewarmed Hank’s solution containing 40 mM ammonium chloride was added into each well and incubated at 37°C for 20 min, after which prewarmed Hank’s solution containing 40 mM ammonium chloride was washed out. This step was not required for the hOCT1 and hOCT2 assays. Then, metformin uptake was also initiated through replacement with Hank’s solution containing metformin with and without GB. For this purpose, 10 μM of metformin and 5 or 500 μg/mL of GB were used. A 100, 100, 1 and 10 μM of cimetidine was adjusted for a well-known inhibitor of hOCT1, hOCT2, hMATE1 and hMATE2-K, respectively, rather than GB. In choosing concentrations of cimetidine and metformin, the respective concentration of cimetidine for each transporter was determined considering the IC_50_ value for each transporter reported by Jin et al. (2019). The metformin concentrations were based on previous work by You et al. (2018); Jin et al. (2019). After 10 min of incubation, Hank’s solution was removed and the cells were immediately washed with ice-cold pure Hank’s solution. The cells were lysed with distilled water and harvested by scraping them off in 200 μL of distilled water, after which they were ultrasonicated for 10 s at 4°C. The samples were centrifuged at 12,000 rpm and 4°C for 10 min, and then the supernatant was stored at −80°C until the LC-MS/MS analysis of metformin (You et al., 2018).

### 2.3 Animal treatment

All protocols used in the animal studies were approved by the Institute of Laboratory Animal Resources of Dongguk University_Seoul, Republic of Korea (Approval number: IACUC-2015-044). Male ICR mice (5-weeks-old, weight 20–25 g) were purchased from the Charles River Company (Seoul, Republic of Korea). Upon arrival, all mice were randomized and housed three per cage under strictly controlled conditions (22°C–25°C and 48%–52% relative humidity) with a 12 h light/dark cycle at an intensity of 150–300 Lux. All mice had free accessed to food and water. They were allowed to acclimate for a week prior to inclusion in the experiments.

The mice were randomly assigned into four groups (i.e., the 1 M, 1 MGB, 28 M and 28 MGB groups) depending on the treatment period (i.e., 1-day and 28-day).

### 2.4 Effect of GB on pharmacokinetics of metformin

In the pre-treatment period (i.e., 27 days), distilled water as vehicle as a vehicle was orally administered to the 1 M and 1 MGB groups for each of the 27 days. Meanwhile, 50 mg/kg metformin (dissolved in distilled water) and 50 mg/kg metformin with 200 mg/kg GB (dissolved in distilled water) were orally administered to the 28 M and 28 MGB groups, respectively, for each of the 27 days. The pharmacokinetic study was conducted at the 28th day after beginning the treatment.

The pharmacokinetic study was conducted by modifying a previously reported method (You et al., 2021). Beginning 8 hrs before the start of experiment, mice were fasted overnight while still having free access to water. On the experiment day (i.e., on the 28th day), metformin with or without GB was orally administered by oral gavage as follows: 50 mg/kg metformin was orally administered to the 1 M (*n* = 24 for 7 profile set) and 28M (*n* = 28 for 8 profile set) groups, while 50 mg/kg metformin with 200 mg/kg GB was simultaneously administered to the 1 MGB (*n* = 24 for 7 profile set) and 28 MGB (*n* = 27 for 8 profile set) groups. Anesthesia was conducted by intraperitoneal (i.p.) injection of 0.05 mL per kg composing 3:1 mixture of zoletil (i.e., tiletamine 125 mg + zolazepam 125 mg in 5 mL) and rompun (xylazine HCl 23.3 mg in 5 mL) before heart puncture. In heart puncture, 31-gauge needle was used to minimize damage to cardiac and pericardial tissues along the needle track and to keep mice alive for several blood collections. The heart puncture was also conducted within the recommended guideline and the approved protocol as followings: a 0.15 mL of one-time blood sampling volume (Florida Atlantic University Institutional Animal Care and Use Committee, 2021), a 1.8 mL of blood sampling as total volume when multiple blood sampling (Diehl et al., 2001), and approximately three times of heart puncture (Golde et al., 2005) in mouse with 20–25 g body weight are recommended. An approximately 120 µL of blood sample was collected *via* heart puncture at 0 (to serve as a control), 5, 15, 30, 60, 120, 180, 240, 360 or 480 min after oral administration of each drug. As three blood samples were obtained per mouse and 24–28 mice were used in total, seven or eight sets of pharmacokinetic data were produced in each group. Insulin syringes coated with heparin (20,000 IU/20 mL) were used for blood sampling. Blood samples were immediately centrifuged at 9,000 rpm and 4°C for 1 min, and a 50 µL aliquot of plasma was collected from the supernatant of each blood sample. At the end of the experiment (24 h), each metabolic cage was rinsed with 5 mL of distilled water, and the rinsing was combined with the 24-h urine sample. After the exact volume of the combined urine sample had been measured, a 50 µL aliquot of the combined urine sample was collected from each mouse. At this time, each mouse was sacrificed by cervical dislocation. The abdomen was opened, and then the entire gastrointestinal tract (GI, including its contents and feces) was removed and transferred into a beaker. The GI was cut into small pieces and 20 mL of methanol was added. After stirring with a glass rod for 1 min, 50 µL of the supernatant was collected from each beaker. All biological samples of plasma, urine and GI were stored at −70°C until being used for the LC-MS/MS analysis of metformin (You et al., 2018).

### 2.5 Effect of GB on metformin distribution to liver and kidneys

In the same way as the pharmacokinetic study, the pre-treatment of metformin with and without GB were administered to the 1 M, 1 MGB, 28 M and 28 MGB groups. On the experiment day (on the 28th day), 50 mg/kg metformin was orally administered to the 1 M and 28 M groups whereas 50 mg/kg metformin with 200 mg/kg GB was orally administered to the 1 MGB and 28 MGB groups. At 1 or 4 h after oral administration, whole blood was collected into the abdominal aorta under the anesthesia, and then 0.9% NaCl-solution was sufficiently perfused through the hepatic portal vein to remove blood form the tissues and to measure the metformin concentration in tissues accurately (Hu et al., 2021; You et al., 2021 ). Mice were sacrificed by loss of blood and cervical dislocation. After centrifugation of the collected blood, 50 µL of plasma was collected from each blood sample. Liver and kidneys were excised, and approximately 1 g of each tissue was weighted. A 4-fold volume of normal saline was added to each tissue, which was homogenized and centrifuged at 12,000 rpm and 4°C for 10 min. Then, 50 µL of the supernatant of each tissue was collected. All collected samples were stored at −80°C until LC-MS/MS analysis of metformin (You et al., 2018).

### 2.6 Effect of GB on mRNA levels of OCTs and MATEs in liver and kidneys

Control, 1 M, 1 MGB, 28 M and 28 MGB groups were assessed to measure the mRNA levels of OCT1, OCT2, MATE1 and MATE2-K in liver and kidneys. Distilled water as vehicle was orally administered to the control group for 28 days. In the other groups, the treatment method was the same as that described in the pharmacokinetic study.

After excising liver and kidneys, total cellular RNA was isolated using a Trizol RNA extraction kit (Thermo Fisher Scientific, Waltham, USA) according to the manufacturer’s instructions. Briefly, total RNA (1 μg) was converted to cDNA using 200 units of reverse transcriptase and 500 ng oligo-dT primers in 50 mM Tris-HCl (pH 8.3), 75 mM KCl, 3 mM MgCl_2_, 10 mM dithiothreitol, and 1 mM dNTPs at 42°C for 1 h. The reaction was then stopped by incubating the reaction at 70°C for 15 min, after which 1 µL cDNA mixture was used as template for PCR amplification. PCR reactions were performed using 1 μL cDNA and 9 μL master mix containing iQ SYBR Green Supermix (Bio-Rad), 5 pmol of forward primer, and 5 pmol reverse primer, in a CFX384 Real-Time PCR Detection System (Bio-Rad). The reaction conditions were 3 min at 95°C followed by 40 cycles of 10 s at 95°C and 30 s at 55°C, after which the plate was read. Fluorescence signal generated with SYBR Green I DNA dye were measured during annealing steps. The specificity of the amplification was confirmed using a melting curve analysis. Data were collected and recorded by CFX Manager Software (Bio-Rad) and expressed as a function of the threshold cycle (CT). The relative quantity of the gene of interest was then normalized to the relative quantity of GAPDH (ΔΔCT). The mRNA abundance in the sample was calculated using equation 2-(ΔΔCT). The following specific primer sets were used (5′to 3′): mouse–β-actin: TCA​AGA​TCA​TTG​CTC​CTC​CTG (forward), GCT​CAG​TAA​CAG​TCC​GCC​TAG (reverse); mouse–Oct1: TGG​AGC​AAA​TTG​CAC​AGA​AG (forward), GGT​CTG​CAA​AC-GAA​GGA​CTC (reverse); mouse–Oct2: AAA​TGG​TCT​GCC​TGG​TCA​AC (forward), TCC​AGC​CAG​ATG​TCA​GTG​AG (reverse); mouse–Mate1: TCC​TTC​CTG​CAA​CTG​GCT​AT (forward), ACT​CCA​CCA​TGC​CAA​GGA​TA (reverse); mouse–Mate2: AGC​TGG​GCT​AAA​AAG​CAA​CA (forward), CCA​GTC​TGG​CTC​TCT​GGT​CT (reverse). Gene specific primers were custom-synthesized by Bioneer (Daejeon, Republic of Korea).

### 2.7 Effect of GB on plasma protein binding of metformin

The plasma protein binding values of metformin with or without GB were measured using a Rapid Equilibrium Dialysis (RED) system (Thermo Fisher Scientific, Rockford, IL, USA). To begin, 300 µL of buffer solution containing 100 mM sodium phosphate and 150 mM sodium chloride, pH 7, was inserted into one chamber of the RED well plate. Meanwhile, 100 µL of mouse plasma containing 1 μg/mL of metformin was inserted into another chamber of the RED well plate. The well plate was then covered with plastic and incubated at 37°C and 50 rpm for 4 h. To measure the effect of GB on the plasma protein binding of metformin, 100 µL of mouse plasma containing 1 μg/mL of metformin with 1 or 100 μg/mL of GB was used. After 4 h incubation, a 50 µL aliquot from each chamber was transferred into a 1.5 mL tube, at which point 500 µL of acetonitrile containing IS (20 ng/mL of carbamazepine) was added into each tube. After being vortexed and centrifugated at 12,000 rpm and 4°C for 10 min, a 5 µL aliquot of the supernatant was injected into the LC-MS/MS system for the analysis of metformin (You et al., 2018). Because metformin was categorized as a positive drug with a low plasma protein binding (Gong et al., 2012; You et al., 2018), 1 μg/mL of donepezil was additionally used as a positive compound as a high plasma protein binding drug (Seltzer, 2005).

### 2.8 LC-MS/MS analysis of metformin and ginsenosides in GB

Metformin was analyzed according to a previously reported method (You et al., 2018). Briefly, 100 μL of acetonitrile containing IS (20 ng/mL carbamazepine) was added to 50 μL of each biological sample. After vortexing and centrifuging a sample at 12,000 rpm for 10 min, 10 µL of the supernatant was analyzed using the LC-MS/MS system. The analytes were monitored using a API4000 triple quadrupole mass spectrometer (AB Sciex, Foster City, CA) equipped with a turbo ion spray interface for electrospray ionization and operated in positive ion mode at 5.5 KV and 500°C. The mass transitions for metformin and IS were m/z 130.1 → 71.0 (collision energy, 31 eV) and 237.2 → 194.1 (25 eV), respectively, in the multiple reaction monitoring (MRM) mode. In the LC system (Thermo Fisher scientific, Seoul, Republic of Korea), the reversed-phase C_18_ column (X-bridge C_18_, 2.1 mm × 100 mm i.d., 3 μm; waters, Ireland) was kept at 4°C. The gradient mobile phase composition was changed from 100% of 0.1% formic acid in water to 100% of acetonitrile for 1.5 min, then switched to 100% of 0.1% formic acid in water for 3.5 min and maintained for 5.5 min at the flow rate of 0.3 mL/min. The retention times of metformin and IS were 1.10 min and 4.08 min, respectively. The detection limit of metformin was 5 ng/mL.

### 2.9 Pharmacokinetic parameters

Standard methods (Gibaldi and Perrier, 1982) were used to calculate the following pharmacokinetic parameters by using non-compartmental analysis (WinNonlin; version 2.1; Scientific Consulting): maximum observed plasma concentration (C_max_), time of maximum observed plasma concentration (T_max_), area under the plasma concentration-time curve from the time of dosing extrapolated to infinity (AUC), renal clearance (CL_R_) and terminal half-life (t_1/2_).

### 2.10 Statistical analysis

A *p*-value < 0.05 was deemed to be statistically significant using a *Student’s t-test* between the two means for the unpaired data or a Tukeyʼs multiple range test of the Social Package of Statistical Sciences (SPSS) posteriori analysis of variance (ANOVA) among the three, four or five means for the unpaired data. The pharmacokinetic parameters were expressed as means ± standard deviations except for T_max_, which was expressed as median (ranges).

### 2.11 Biochemistry analysis

A 24 h urine sample was collected to measure urine output and creatinine (Cr) levels to measure kidney function in the 1 M, 1 MGB, 28 M and 28 MGB groups (*n* = 5 for each group). Blood was collected to measure total protein, albumin, urea nitrogen, glutamate oxaloacetate transaminase, glutamate pyruvate transaminase and creatinine concentration in plasma. Blood samples were analyzed by Green Cross Reference Lab (Seoul, Republic of Korea).

## 3 Results

### 3.1 Effect of GB on metformin uptake in HEK293 cells overexpressing hOCT1, hOCT2, hMATE1 and hMATE2-K

**FIGURE 1:**
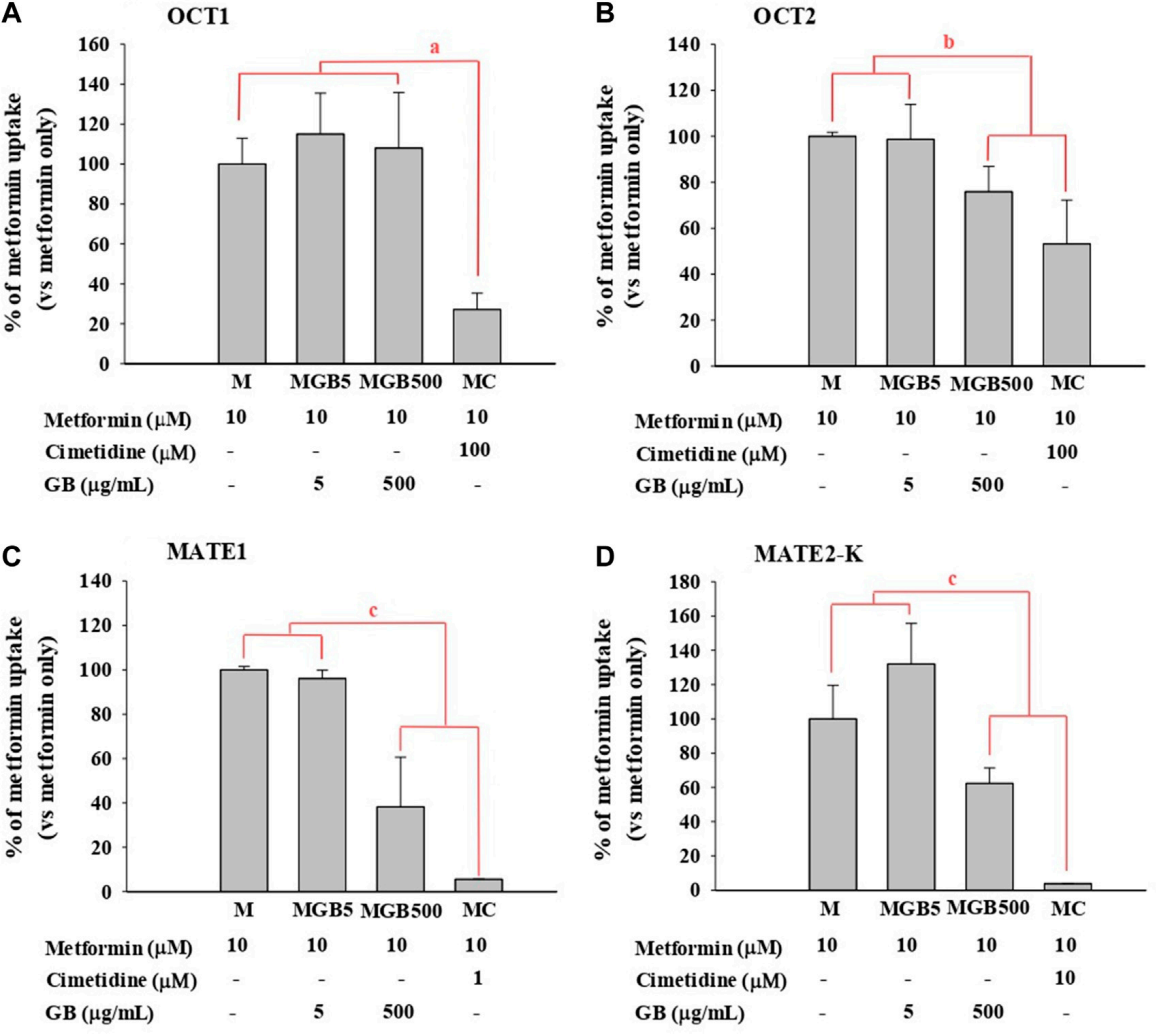
.Metformin uptake in HEK293 cells overexpressing **(A)** hOCT1, **(B)** hOCT2, **(C)** hMATE1, and **(D)** hMATE2-K. Cimetidine at 100, 100, 1 and 10 μM was used as a well-known of OCT1, OCT2, MATE1 and MATE2-K, respectively. ^a^MC group was significantly different (*p* < 0.05) from other groups. ^b^MGB500 and MC groups were significantly different (*p* < 0.05) from M and MGB5 groups. ^c^MGB500 and MC groups were significantly different (*p* < 0.05) from M and MGB5 groups, and also there was a significant difference (*p* < 0.05) between MC and MGB500 groups.

As shown in Figure 1, metformin uptake in HEK293 cells overexpressing hOCT2, hMATE1 and hMATE2-K was significantly reduced (by 24.1, 61.8, and 37.6%, respectively) by 500 μg/mL of GB. However, 5 μg/mL of GB did not change metformin uptake in any of the groups.

As a well-known inhibitor against each transporter, cimetidine significantly reduced metformin uptake in HEK293 cells overexpressing hOCT1, hOCT2, hMATE1 and hMATE2-K (by 72.9%, 46.8%, 94.5% and 96.2%, respectively).

### 3.2 Effect of GB on metformin pharmacokinetics

**FIGURE 2:**
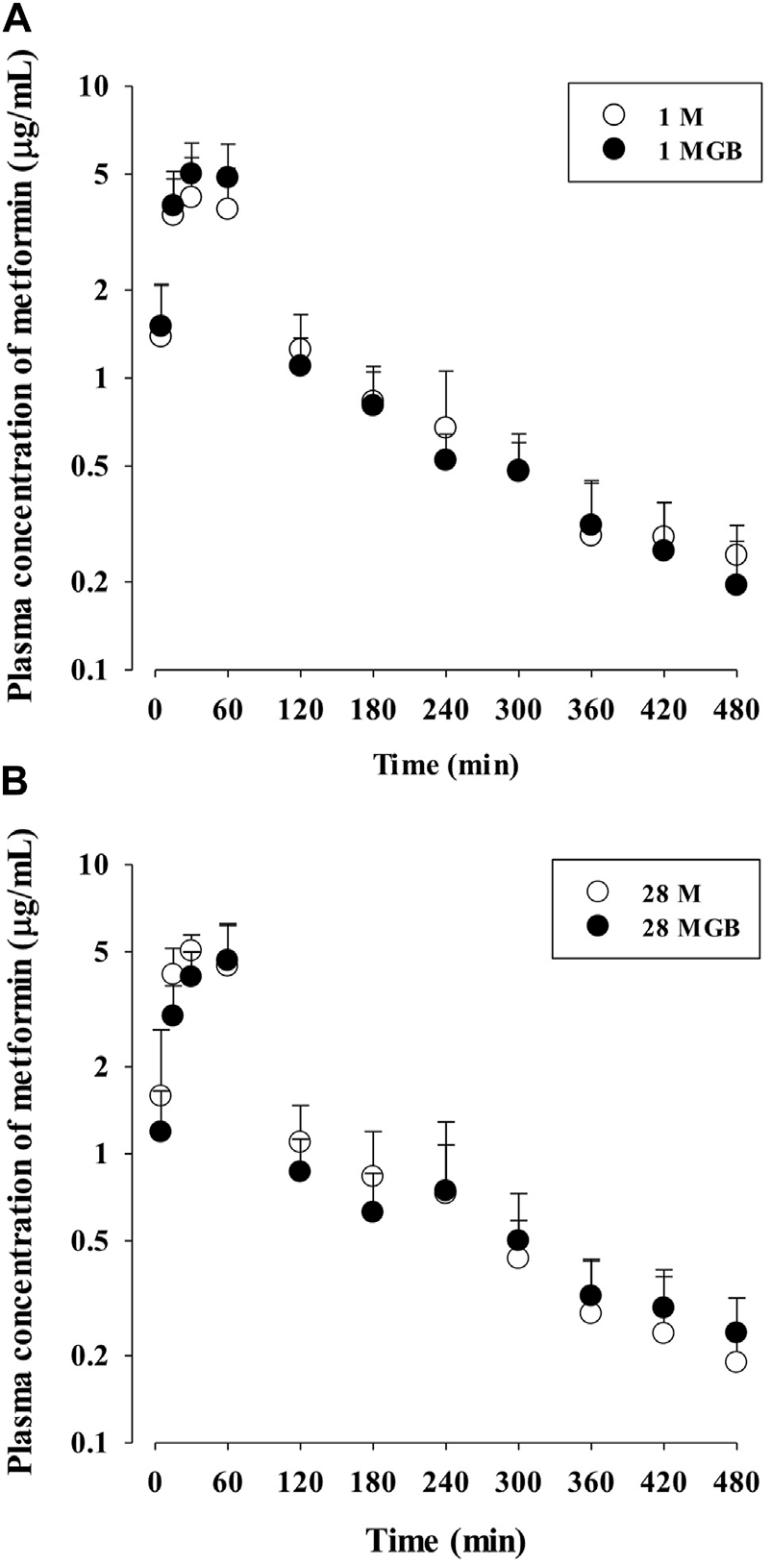
The mean plasma concentration-time profiles of metformin after oral administration of metformin with (●; 1 MGB and 28 MGB) and without GB (○; 1 M and 28 M) to mice, respectively. The doses of metformin and GB were 50 and 200 mg/kg, respectively. Treatment periods were 1-day **(A)** and consecutive 28-day **(B)**, respectively.

The mean arterial plasma concentration-time profile of metformin after its oral administration with and without GB is shown in Figure 2, and some relevant pharmacokinetics parameters are listed in Table 1. There was no change on any pharmacokinetic parameters of metformin among the four groups.

**TABLE 1:** Mean (±SD) pharmacokinetic parameters of metformin after its oral administration with or without GB in 1 M, 1 MGB, 28 M, and 28 MGB groups, respectively. The doses of metformin and GB were 50 and 200 mg/kg, respectively.

	1 M (*n* = 7)	1 MGB (*n* = 7)	28 M (*n* = 8)	28 MGB (*n* = 8)
Body weight (g)^a^	25.0 ± 2.59	26.9 ± 1.82	27.5 ± 1.18	27.5 ± 0.950
AUC (μg min/mL)	575 ± 110	615 ± 87.2	567 ± 99.5	535 ± 67.8
C_max_ (μg/mL)	4.45 ± 1.36	5.46 ± 1.32	5.54 ± 0.799	4.50 ± 1.23
T_max_ (min)^b^	30 (15–60)	30 (30–60)	30 (15–60)	30 (30–60)
T_1/2_ (min)	108 ± 51.7	140 ± 78.9	78.9 ± 15.8	116 ± 58.4
CL/*F* (mL/min/kg)	89.7 ± 17.1	82.7 ± 12.0	90.6 ± 15.7	94.8 ± 11.4
CL_R_/*F* (mL/min/kg)	57.7 ± 13.8	55.9 ± 11.8	54.3 ± 16.9	61.3 ± 18.0
Ae (% of dose)	65.6 ± 16.6	68.2 ± 15.1	60.3± 20.3	64.7 ± 16.7
GI (% of dose)	2.46 ± 1.26	2.80 ± 1.07	1.49± 1.12	2.17 ± 0.698

^a^ 1 M and 1 MGB, groups were significantly different (*p* < 0.05) from 28 M to 28 MGB, groups.

^b^ Data are median (range).

### 3.3 Effect of GB on metformin distribution in the livers and kidneys

Metformin concentrations in plasma, livers and kidneys, along with their T/P ratios at 1 and 4 h, are shown in Table 2. In the livers, metformin concentration in the livers of 28 MG B group at 4 h was significantly higher (by 37.3, 59.3% and 60.9% *versus* 1 M, 1 MGB and 28 M, respectively) than other three groups. There was no difference on liver concentrations of metformin at 1 h among the four groups. The T/P ratios of metformin at 1 and 4 h in the livers also were changed among the four groups.

**TABLE 2:** Mean (±SD) metformin concentrations (μg/mL for plasma and μg/g tissue) and its tissue to plasma (T/P) ratios at 1 and 4 h in the livers and kidneys of 1 M, 1 MGB, 28 M, and 28 MGB groups, respectively.

		1 M (*n* = 5)	1 MGB (*n* = 5)	28 M (*n* = 5)	28 MGB (*n* = 5)
Plasma					
1 h	Concentration (μg/mL)	3.43 ± 0.230	3.48 ± 0.625	3.52 ± 0.624	3.01 ± 0.597
4 h	Concentration (μg/mL)	0.591 ± 0.132	0.506 ± 0.191	0.501 ± 0.180	0.502 ± 0.0979
Livers					
1 h	Concentration (μg/g liver)	16.9 ± 4.96	15.3 ± 4.93	14.6 ± 4.06	19.8 ± 2.40
	T/P	(4.96 ± 1.51)	(4.68 ± 2.50)	(4.39 ± 1.96)	(6.73 ± 1.06)
4 h	Concentration (μg/g liver)^a^	5.79 ± 1.15	4.99 ± 0.765	4.94 ± 0.892	7.95 ± 1.22
	T/P	(10.4 ± 4.39)	(11.9 ± 7.15)	(10.8 ± 3.72)	(16.3 ± 3.57)
Kidneys					
1 h	Concentration (μg/g kidney)	33.9 ± 15.1	28.4 ± 6.94	29.4 ± 7.68	22.3 ± 2.16
	T/P	(9.87 ± 4.17)	(8.16 ± 1.31)	(8.37 ± 1.67)	(7.68 ± 1.82)
4 h	Concentration (μg/g kidney)	16.4 ± 0.818	15.2 ± 1.21	12.2 ± 2.23	11.9 ± 2.04
	T/P	(28.9 ± 6.56)	(35.6 ± 19.2)	(25.6 ± 4.56)	(24.8 ± 8.07)

The values in parenthesis represent the tissue to plasma (T/P) ratios.

^a^ 28 MGB, was significantly different (*p* < 0.05) from other groups.

In the kidneys, there were no difference of metformin concentrations and T/P ratios among the four groups.

### 3.4 Effects of GB on mRNA levels of OCT1, OCT2, MATE1 and MATE2-K in liver and kidney

**FIGURE 3:**
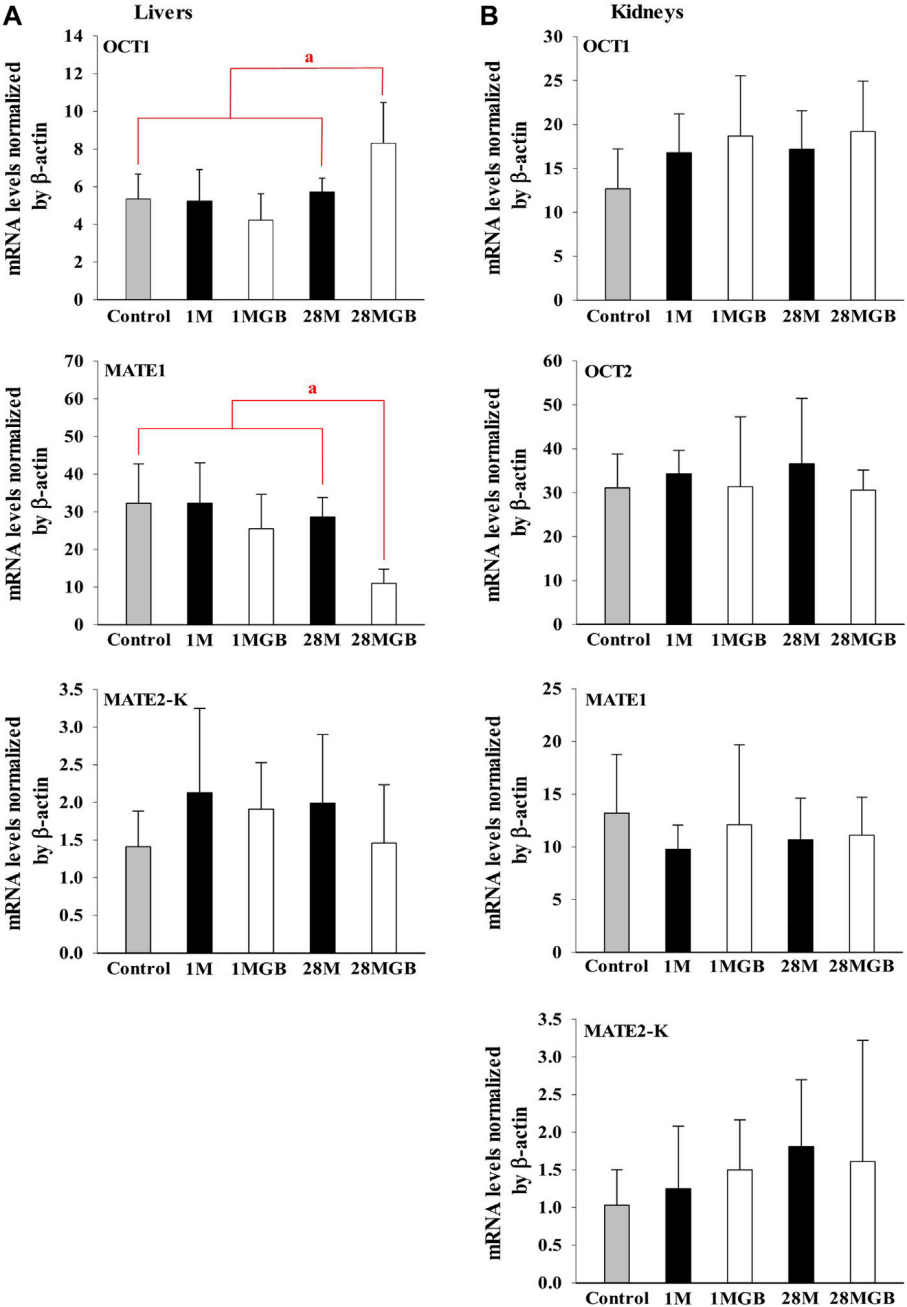
Relative mRNA expressions of OCT1, OCT2, MATE1 and MATE2-K in **(A)** livers and **(B)** kidneys of control, 1 M, 1 MGB, 28 M and 28 MGB groups, respectively. ^a^28 MGB was significantly different (*p* < 0.05) from other groups.

The mRNA levels of OCT1, OCT2, MATE1 and MATE2-K in each of the liver and kidneys were measured in the control, 1 M, 1 MGB, 28 M and 28 MGB groups, and the results are shown in Figure 3. In the livers, the mRNA level of OCT1 in 28 MGB group was significantly increased (by 55.4%, 58.4%, 96.5%, and 44.6%) compared to control, 1 M, 1 MGB, and 28 M, respectively. On the other hands, the mRNA level of MATE2-K in 28 MGB group was significantly decreased (by 66.0%, 66.0%, 56.9%, and 61.6%) compared to control, 1 M, 1 MGB, and 28 M, respectively. There was no difference of mRNA level of MATE2-K in the livers among the four groups, and the mRNA level of OCT2 was not detected in the livers of any groups.

In the kidneys, no changes were observed in the mRNA levels of any transporters in any groups.

### 3.5 Effect of GB on plasma protein binding of metformin

The plasma protein binding values of metformin with or without GB were as follows: 15.9% ± 6.54%, 14.3% ± 5.47% and 17.3% ± 4.17% with metformin alone, metformin with 1 μg/mL GB, and metformin with 100 μg/mL GB, respectively (*n* = 3 for each group). There were no significant difference between any of these values. For validation, the plasma protein binding value of 1 μg/mL donepezil as a high plasma protein binding drug was 81.2%, which was similar as the previously reported value (Seltzer, 2005).

### 3.6 Effect of GB on livers and kidney functions

The body weight, biochemical profiles, and parameters for liver and kidney function for the 1 M, 1 MGB, 28 M and 28 MGB groups are presented in Table 3. In all groups, all biochemical profiles and tissue weights (i.e., livers and kidney) were within normal ranges according to the reference values from Mitruka and Rawnsley (1981).

**TABLE 3:** Mean (±SD) body weight, physical chemistry, and weights of liver and kidneys in 1 M, 1 MGB, 28 M and 28 MGB groups, respectively.

	1 M (*n* = 5)	1 MGB (*n* = 5)	28 M (*n* = 5)	28 MGB (*n* = 5)
Body weight (g)	23.3 ± 5.26	24.5 ± 4.95	24.6 ± 3.48	25.4 ± 3.79
Plasma				
Total protein (g/dL)	5.10 ± 0.64	4.95 ± 0.84	5.15 ± 0.48	4.90 ± 0.78
Albumin (g/dL)	3.64 ± 0.45	3.82 ± 0.548	3.90 ± 0.51	3.75 ± 0.31
Glucose (mg/dL)	260 ± 49.2	253 ± 21.7	261 ± 31.8	249 ± 52.7
AST (IU/L)	78.3 ± 6.48	80.3 ± 10.2	76.2 ± 4.85	79.3 ± 7.11
ALT (IU/L)	26.5 ± 5.12	27.0 ± 3.99	25.8 ± 5.47	26.9 ± 2.18
Urea nitrogen (mg/dL)	43.5 ± 9.12	42.3 ± 3.48	44.3 ± 3.88	45.1 ± 5.71
Creatinine (mg/dL)	0.51 ± 0.026	0.50 ± 0.031	0.53 ± 0.019	0.52 ± 0.040
Liver weight (% of BW)	2.83 ± 0.12	2.69 ± 0.31	2.70 ± 0.25	2.72 ± 0.19
Kidney weight (% of BW)	0.97 ± 0.11	0.98 ± 0.084	0.99 ± 0.057	0.98 ± 0.061

## 4 Discussion

In drug interactions (e.g., drug-drug and drug-herb interactions), treatment period is a critical factor to influence metabolic enzymes and/or transporters, which affects the pharmacokinetic profile of a victim drug (Choi, 2020; Choi and Chin, 2020; Iwatsubo, 2020; Sudsakorn et al., 2020). For example, Ma et al. (2015) reported that consecutive co-treatment of atenolol (specially, 7-, 15-, 30-, and 60-day treatment) reduced the renal excretion of metformin by the downregulation of MATE1 in kidneys, and consequently increased plasma concentrations of metformin. However, single co-treatment of atenolol with metformin did not change the pharmacokinetics of metformin. You et al. (2018) reported that 28-day treatment of *Houttuynia cordata* extract with metformin improved the glucose tolerance of metformin, because *H. cordata* extract reduced MATE1-mediated metformin efflux from the livers and consequently increased metformin concentration in the livers. In another study, the results for the combination of *Scutellariae Radix* extract and metformin also showed that only 28-day co-treatment of *Scutellariae Radix* extract altered the pharmacokinetics of metformin (Yim et al., 2017). These cases support that the treatment period of combination therapy can cause dramatically different outcomes in a victim drug’s pharmacokinetic characteristics. Thus, we designed the experiments to evaluate the treatment period effect (e.g., 1- and 28-day) of GB on metformin pharmacokinetics.

In general, the change in the systemic exposure of a victim drug serves as primary evidence of the occurrence of pharmacokinetic interaction in HDIs (Choi, 2020). Systemic exposure is typically evaluated depending on AUC fold-change (Jin et al., 2019): if the AUC of a victim drug is increased, it might be attributable to an increase in absorption, a decrease in tissue distribution, or the elimination of a victim drug by combined treatment of herb. If there is no change in the AUC of a victim drug, HDI can be regarded as absent. On the other hand, the erroneous interpretation of systemic exposure change has been reported in drug interactions (Ma et al., 2015; Yim et al., 2017; Choi et al., 2018; You et al., 2018; Choi, 2020; Choi and Chin, 2020; Sanz Codina and Zeitlinger, 2022; Yu and Wang., 2022), because drug concentrations in tissue as opposed to overall systemic exposure of the drug can contribute to pharmacological and/or toxicological activities. Therefore, it is highly recommended to assess both systemic exposure and tissue distribution *in vivo* of the drug to evaluate or predict drug interactions (Choi, 2020; Kimoto et al., 2022; Zang et al., 2022).

The OCTs and MATEs are main transporters to trigger metformin movement in the body, which is associated with metformin efficacy or toxicity (Stage et al., 2015; Storelli et al., 2022). Metformin concentration in the livers is determined by transporter-mediated metformin uptake and efflux, because metformin metabolism is negligible. The OCT1 in sinusoidal membrane uptakes metformin from blood to hepatocytes, after which MATE1 in canalicular membrane effluxes metformin into bile (i.e., biliary excretion) (Burt et al., 2016; Li et al., 2022). In the proximal tubules of kidneys, OCT2 in sinusoidal membrane uptakes metformin from blood into proximal tubules and then MATE1 and MATE2-K in the apical membrane transports metformin from proximal tubules into urine. Renal excretion is a main elimination route of metformin, and metformin transport among blood, proximal tubules and urine governs systemic exposure (i.e., the AUC and metformin concentration in blood) (Scotcher et al., 2020; Krishnan et al., 2022). Overall, metformin concentrations in the blood as well as liver and kidneys are associated with efficacy (i.e., inhibition of gluconeogenesis in liver) and toxicity (i.e., lactic acidosis and fluid retention) (You et al., 2018; Chae et al., 2019; Krishnan et al., 2022). In line with this view, it is necessary to investigate whether GB affects both systemic exposure and tissue concentration of metformin in liver and kidneys considering the change of efficacy or toxicity along with the pharmacokinetic changes.

First, the effect of GB on *in vitro* transporter-mediated metformin transport can be different from their changes in *vivo* system. In our results, the *in vivo* change of metformin transports by GB (Tables 1, 2) were not the same as the *in vitro* inhibitory effect of GB (Figure 1), probably due to the different effect of GB on OCTs and MATEs-mediated metformin transporters. It was reported that the percentages of ginsenosides-Rb1, -Rb2, -Rc, -Rd, -Re, -Rg1, -R-Rg2, -S-Rg2, -R-Rg3 and -S-Rg3 in GB were 1.29, 2.56, 0.86, 3.30, 6.50, 0.24, 0.47, 0.75, 0.14% and 0.36%, respectively, in our previous paper (Han and Choi, 2020). In Figure 1, 500 μg/mL of GB significantly inhibited metformin uptake (by 46.8, 61.8% and 37.6% *via* OCT2, MATE1 and MATE2-K, respectively) compared to metformin group. This result proposed that 6.45, 12.8, 4.30, 16.6, 32.5, 1.20, 2.35, 3.75, 0.70 and 1.80 μg/mL of ginsenosides-Rb1, -Rb2, -Rc, -Rd, -Re, -Rg1, -R-Rg2, -S-Rg2, -R-Rg3 and/or -S-Rg3 in 500 μg/mL GE (in the highest concentration GB used in *vitro* study) might be involved to inhibit MATE1 or MATE2-mediated metformin uptake in this *in vitro* study. As a view for *in vivo* exposure of ginsenosides, only ginsenosides-Rb1, -Rc, -Rd and -Re were measured in plasma of 1 MG B and 28 MG B group (*n* = 3 for each group as the preliminary study). In 1 MG B group, the mean *C*_max_ values of ginsenoside-Rb1, -Rc, -Rd and -Re were 0.252 ± 0.108, 0.201 ± 0.0684, 0.782 ± 0.247 and 0.654 ± 0.301 μg/mL, respectively. In the livers of 1 MG B group, only ginsenosides-Rd and -Re were detected with their concentration ranges were 0.239–0.684 μg/mL and 0.523–2.48 μg/mL, respectively. Although 500 μg/mL of GB directly inhibited OCT2, MATE1 and MATE2-K mediated metformin uptake *in vitro* (Figure 1), there was no change of metformin concentrations in the plasma, liver and kidneys (Tables 1, 2 ; Figure 2) and mRNA expressions of OCT1, OCT2, MATE1, or MATE2-K *in vivo* in the livers and/or kidneys of 1 MG B group (Figure 3). These results indicated that 200 mg/kg GB administration might not enough to directly inhibit transporter-mediated metformin movement *in vivo*. As possible reasons, the lower concentrations of ginsenosides-Rd and Re in the livers than those contained in 500 μg/mL GB can be considered. In addition, it might be deduced that the concentrations of other unknown compounds of GB, 83.5% of GB, and their direct inhibitory activities against OCT1, MATE1, and/or MATE2-K in the livers of 1 MG B group were different from those in *vitro* 500 μg/mL GB treatment, although the 83.5% of the constituents’ concentrations in GB could not be measured. In addition, HEK293 cells overexpressing one of OCT1, OCT2, MATE1 and MATE2-K are commercial products of cryopreserved transporter cells. These cells transiently overexpress each single human transporter protein, and they can be used to conveniently evaluate transporter-mediated drug interactions regardless of their expressed tissues (Burt et al., 2016; Sudsakorn et al., 2020; Mathialagan et al., 2021). In HEK293 cells individually overexpressing OCT1, OCT2, MATE1 or MATE2-K, the high concentration of GB (500 μg/mL, not 5 μg/mL) substantially inhibited OCT2, MATE1 and MATE2-K-mediated metformin uptake: the magnitude of the inhibitory effect of GB, which was evaluated in individual transporters, was found to be highest in MATE1 and MATE2-K, followed by OCT2 (Figure 1). However, different from *in vitro* individual transporter-mediated metformin uptake, it might be attributed that *in vivo* OCTs and MATEs operate metformin transport together, and they diversely regulate metformin transport according to their expressed membrane sides and/or tissues (Krishnan et al., 2022; Storelli et al., 2022). If the distributions (i.e., consequent concentrations) of any components in GB are diverse in the livers and kidneys, they can produce different inhibitory effect on the transporters between the livers and kidneys (Han and Choi, 2020). For example, the mRNA level of OCT1 was increased and that of MATE1 was reduced in the livers of 28 MG B group compared to other four groups, whereas there was no change of any mRNA levels of OCT1, OCT2, MATE1, and MATE2-K in the kidneys of all groups (Figure 3 ).

Secondly, GB co-treatment differently changed metformin concentration in the tissues and blood. The 28-day co-treatment of GB significantly increased metformin concentration in the liver of the 28 MGB group compared to that in the 28 M group (Table 2), probably due to the changed mRNA levels of OCTs and MATEs in the liver (Figure 3): the increase of OCT1-mediated metformin uptake into hepatocyte and the decrease of MATE1-mediated metformin efflux into bile might cause the higher metformin concentration in the liver of the 28 MGB group than the 28 M group, as was similarly described in previous reports (Ma et al., 2015; You et al., 2018). However, GB did not affect any AUC values of metformin (i.e., systemic exposure of metformin) in any groups (Table 1) due to the no alternation of any mRNA levels of OCT1, OCT2, MATE1 and MATE2-K in the kidneys of any groups (Figure 3). The metformin distribution in kidneys and its renal excretion mainly determine the systemic exposure. In particular, OCT2 is the most predominant transporter in metformin uptake into kidneys and OCT2-mediated metformin uptake might contribute more substantially to the renal concentration of metformin than MATE1 and MATE2-K-mediated metformin (Motohashi and Inui, 2013; Burt et al., 2016). Thus, the unchanged metformin concentrations in the kidneys and subsequently unchanged systemic exposure of metformin (Tables 1, 2) can be explained based on the no alternation of any mRNA level of OCT1, OCT2, MATE1 and MATE2-K in the kidneys of any groups (Figure 3).

Third, treatment period is an important factor to cause transporter-mediated drug interactions. Aside from 28-day co-treatment of GB and metformin, 1-day co-treatment of GB did not affect the mRNA levels of OCT1 and MATE1 in liver or kidneys (Figure 3) or metformin concentrations in plasma, liver and kidneys (i.e., systemic exposure and local exposure in the tissues) (Table 1, 2). In tissue distribution, 28-day co-treatment of GB with metformin increased metformin concentration *via* OCT1 induction and MATE1 inhibition in the livers, a pharmacological target tissue. Similar phenomena were reported by Yim et al. (2017) and You et al. (2018): long-term treatment of herbal products changed the mRNA levels of OCT1 and/or MATE2-K-mediated metformin transport in liver.

Forth, the pharmacokinetic change of metformin by GB can be associated with the efficacy change of metformin. Changes in metformin concentration in the liver, as its pharmacological target tissue, might be related to enhancing metformin’s efficacy. Chae et al. (2019) suggested that the increased concentration of metformin in the liver is a possible reason to improved metformin efficacy such as enhancing glucose tolerance and ameliorating insulin resistance and hepatic steatosis in metformin plus GB combination compared to metformin single treatment. The following phenomena were observed: the significantly higher concentration (by 83.6%) of metformin in the livers along with the reduced AUC_glucose_ (by 29.3%) and reduced HOMA-IR (by 15.4%) values in mice fed high-fat diet with metformin plus GB for 98 days compared to those in mice fed with fed high-fat diet with metformin for 98-day. This could be due to an increase of OCT-mediated metformin uptake into liver and the subsequent increase in AMP-activated protein kinase together with co-treated GB. Thus, it is plausible that GB changes metformin distribution in the liver without systemic exposure alternation. Although the efficacy change of metformin by GB co-treatment was not investigated at this time, metformin’s efficacy can be improved by 28-day co-treatment of GB, in accordance with the increased metformin concentration in the liver as the similar result of Chae et al. (2019) . Moreover, the toxicity of metformin did not arise with GB co-treatments ( Table 3 ). Lactic acidosis is one of the major metformin toxicities, and it is associated with metformin’s systemic exposure (Mathialagan et al., 2021). The lack of changes in metformin concentration in the blood in all groups indicates that additional metformin toxicity was not observed.

## 5 Conclusion

We can suggest that the change in metformin pharmacokinetics by GB co-treatment can contribute to the changes in efficacy and toxicity. In particular, these results indicate that systemic exposure and tissue distribution depending on treatment periods may be important in HDIs, although further study is needed to clarify the mechanism involved in the transcriptional or transcriptional regulation of MATE1 expression in the liver depending on treatment periods. Although the mechanism of metformin and GB combinational effect change was not proved, the pharmacokinetic change of metformin by 28-day co-treatment of GB can be suggested.

## Data Availability

The original contributions presented in the study are included in the article/supplementary material, further inquiries can be directed to the corresponding author.
